# Lessons learned from human HIV vaccine trials

**DOI:** 10.1097/COH.0000000000000362

**Published:** 2017-03-27

**Authors:** Justin Pollara, David Easterhoff, Genevieve G. Fouda

**Affiliations:** a Department of Surgery; b Duke Human Vaccine Institute, Duke University School of Medicine, Durham, North Carolina, USA

**Keywords:** B-cell repertoire, broadly neutralizing antibodies, vaccine

## Abstract

**Purpose of review:**

The ability to induce broadly neutralizing antibody (bNAb) responses is likely essential for development of a globally effective HIV vaccine. Unfortunately, human vaccine trials conducted to date have failed to elicit broad plasma neutralization of primary virus isolates. Despite this limitation, in-depth analysis of the vaccine-induced memory B-cell repertoire can provide valuable insights into the presence and function of subdominant B-cell responses, and identify initiation of antibody lineages that may be on a path towards development of neutralization breadth.

**Recent findings:**

Characterization of the functional capabilities of monoclonal antibodies isolated from a HIV-1 vaccine trial with modest efficacy has revealed mechanisms by which non-neutralizing antibodies are presumed to have mediated protection. In addition, B-cell repertoire analysis has demonstrated that vaccine boosts shifted the HIV-specific B-cell repertoire, expanding pools of cells with long third heavy chain complementarity determining regions – a characteristic of some bNAb lineages.

**Summary:**

Detailed analysis of memory B-cell repertoires and evaluating the effector functions of isolated monoclonal antibodies expands what we can learn from human vaccine trails, and may provide knowledge that can enable rational design of novel approaches to drive maturation of subdominant disfavored bNAb lineages.

## INTRODUCTION

Despite recent advances in HIV-1-prevention strategies, there remain over 2 million new HIV-1 infections each year [1–4]. Therefore, an effective HIV vaccine is needed in order to abrogate new infections and reach the target of ending the global AIDS epidemic by the year 2030. There have been six human HIV-vaccine efficacy trials conducted to date [5]. Only one – the RV144 Thai trial – has demonstrated any evidence of vaccine-mediated protection, with a modest estimated vaccine efficacy of 31% [6]. Similar to most licensed vaccines [7], a comprehensive analysis of the immune correlates of reduced infection risk in the RV144 trial identified multiple aspects of vaccine-induced humoral immune responses as contributing to reduced risk of infection [5,8–10,11^▪^]. Unexpectedly, non-neutralizing antibodies capable of mediating Fc-dependent antiviral effector functions were identified as a correlate of reduced infection risk (see review by C. Moog [12]) [8,13]. The limited success of the RV144 trial demonstrated the proof of principle that vaccination can impart protection from HIV-1 infection, and provides a foundation for development of the next generation of candidate vaccines designed for improved effectiveness in diverse and higher-risk populations. Passive protection studies conducted in nonhuman primate model systems have provided evidence that broadly neutralizing antibodies (bNAbs), defined as those capable of neutralizing multiple difficult to neutralize (tier 2 [14]) HIV-1 primary isolates, are highly effective in preventing infection [15^▪^,^16^,^17^]. These results suggest that inducing bNAb responses by vaccination will be required to improve upon the results of the RV144 clinical trial and to develop a highly effective global HIV vaccine.

Although bNAb responses have not yet been observed in human HIV-1 vaccine trials, recent data from the RV144 and RV305 clinical trials suggest that antibody lineages with long third heavy chain complementarity determining regions (HCDR3s), a characteristic associated with some bNAb lineages [18], were initiated by the vaccine regimen [19]. In this review, we summarize studies of antibody effector functions that have been induced to date with experimental HIV vaccines, and speculate on what may be needed to achieve improved vaccine efficacy.

**Box 1:**
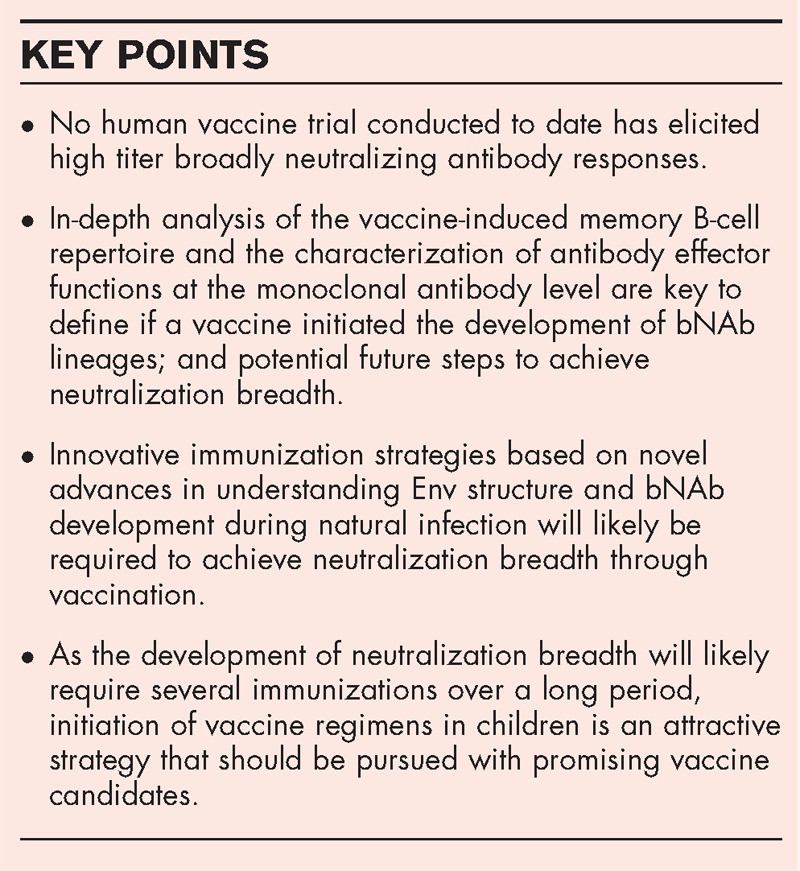
no caption available

## BROADLY NEUTRALIZING ANTIBODY RESPONSES HAVE NOT BEEN INDUCED BY ANY CANDIDATE VACCINE TESTED IN HUMAN CLINICAL TRIALS

There have been six HIV-vaccine efficacy trials [5,11^▪^], and a recent search of clinicaltrials.gov (search term: HIV vaccine; intervention: biological; search date: 2 November 2016) identified over 400 completed human clinical studies of candidate HIV vaccines. A general overview of these studies indicated the evaluation of diverse assortments of vaccine regimens, vectors, routes of vaccine administration, adjuvants, immunogens, and study populations (many strategies recently reviewed in [10]). These clinical trials were themselves preceded and informed by preclinical studies in various animal models, further multiplying the number of studies and varieties of approaches that have been conducted in search of an effective vaccine to prevent HIV. Despite this robust and earnest effort, there have been no publications describing induction of HIV-1 antibody responses capable of broad neutralization by a human vaccine trial. The VAX004 trial provided the only evidence of tier 2 virus neutralization, but at low titer and only in a subset of vaccine recipients [20]. Thus, from the human HIV vaccine trials conducted to date, detectable bNAb responses are difficult to induce by vaccination. Nonetheless, careful analysis of these unsuccessful efforts and of bNAbs generated during natural infection is necessary to inform vaccine design.

## UNIQUE CHARACTERISTICS OF HIV-1 BROADLY NEUTRALIZING ANTIBODIES IMPEDE THEIR INDUCTION THROUGH VACCINATION

The neutralizing breadth observed for plasma or serum antibodies generated during natural infection has been studied by isolation of individual monoclonal antibodies (mAbs). bNAbs can be detected in up to 50% of HIV-1-infected individuals, but these responses are subdominant to those lacking neutralizing breadth and develop only after several years of exposure to replicating virus [21–25]. In rare cases, HIV-infected individuals can develop a dominant bNAb response with the ability to overcome the vast genetic diversity and glycan-shielding that characterize the HIV-1 envelope protein (Env) [26,27,28^▪▪^]. Antibodies with the greatest breadth target conserved regions of neutralization vulnerability on the HIV envelope trimer: the CD4-binding site (CD4bs), the variable region 1 and 2 (V1/V2) glycan, the variable region 3 (V3) glycan, the gp120–gp140 interface, and the gp41 membrane proximal external region [29^▪▪^]. Significant advances have recently been made in identifying common immunogenetic characteristics of bNAbs – extensive somatic hypermutation, polyreactivity or autoreactivity, and atypically long heavy chain complementarity determining region 3 (HCDR3) or short CDRL3s – all of which are normally restricted by immune tolerance mechanisms and therefore uncommon in the B-cell repertoire [21,29^▪▪^]. The collective observations from natural HIV infection (reviewed in more detail by A. McKnight [30]) illuminate the underlying reasons why human HIV vaccine trials have yet to induce bNAb responses.

## WHAT SHOULD WE EXPECT TO LEARN FROM PAST AND CURRENT HUMAN HIV VACCINE STUDIES?

Given the slow development, rarity, and subdominance of bNAb responses in natural HIV-1 infection, it is unsurprising that vaccine-recipient plasma or serum samples with broad HIV-1-neutralizing activity have not yet been induced by candidate vaccines. However, this observation is not sufficient to rule out the possibility that subdominant bNAb responses were induced, or more likely, whether the vaccine has stimulated expansion of B cells that produce bNAb precursors. Detailed characterization of the vaccine-induced B-cell response is critical for identifying strategies that could be used to further drive maturation and expansion of early bNAb responses through subsequent vaccination. The techniques required to identify early potential bNAb lineages or subdominant responses in vaccine recipients include antigen-specific B-cell isolation or total B-cell isolation for memory B-cell culture, sequencing and deep sequencing of immunoglobulin heavy and light chain genes, and production of mAbs by recombinant production methods. These techniques are resource and labor-expensive, and require considerable time and expertise (contribution of D. Corti [31]). Thus, practical constraints have limited the widespread deployment of this level of analysis to only a subset of human vaccine trials, and to only a small subset of vaccine recipients within these trials. Regardless of these limitations, application of B-cell repertoire analysis to vaccine recipients enrolled in the RV144 and RV305 clinical trials has provided new insight into the antigen-specific B-cell responses.

## WHAT HAS BEEN LEARNED FROM B-CELL REPERTOIRE ANALYSIS OF ALVAC-HIV + AIDSVAX B/E GP120 IMMUNIZED HUMANS WITH TIER 1 PLASMA NEUTRALIZATION

The ALVAC-HIV (vCP1521) prime AIDSVAX B/E gp120 boost vaccine regimen used in the RV144 HIV-1 vaccine trial reduced HIV-1 acquisition risk with an estimated vaccine efficacy of 60% at 12 months [32] and 31% at 42 months [6]. Risk of infection correlated inversely with plasma IgG antibody binding of Env V1/V2, and directly with plasma Env-specific IgA levels [8,33]. Neither the polyclonal plasma, nor mAbs isolated from RV144 vaccine recipients had neutralization activity beyond tier 1 viruses. The impact of additional vaccine boosts on the quality and quantity of the vaccine-induced humoral response was evaluated in the follow-up study, RV305.

The RV305 HIV-1 clinical trial was a delayed (6–8 years later) and repetitive boosting of RV144 vaccine -recipients with the same immunogens that were used in the RV144 vaccine regimen. In the primary plasma analysis of RV305, like in the RV144 trial, no heterologous tier 2 neutralizing activity was detected [34,35]. Whereas Env-specific antibody responses rapidly waned after completion of the RV144 vaccine- regimen [36], a longitudinal interrogation of the post-RV144 and post-RV305 vaccine-induced memory B-cell repertoires found that B-cell clonal lineages started in the RV144 vaccine trial could be boosted many years later [19]. These data suggested that even though plasma Env antibody titers were not sustained at high levels, Env vaccination did induce long-lived memory B cells, and possibly memory CD4^+^ T cells, that can be recalled with boosting [37,38^▪^].

The CD4bs, V2-glycan, and V3-glycan bNAbs frequently have a long HCDR3 [18,39,40]. Compared to the post-RV144 Env-specific memory B cells, after RV305 there was a substantial increase in Env-reactive antibodies with long HCDR3s [19]. A subset of the long HCDR3 antibodies isolated after RV305 were CD4bs antibodies with an epitope that overlapped the CD4bs bNAb B12 (Easterhoff *et al.*, [41^▪▪^]). Structural analysis of one of these antibodies found preferential binding to open Env trimers, thus limiting their ability to neutralize more difficult-to-neutralize isolates with closed trimers. Whereas the CD4bs antibodies isolated after RV305 were not bNAbs, these data reiterate that immunizing with Env protein induces a spectrum of responses from easy-to-induce dominant responses to more difficult-to-induce subdominant responses, and that repetitive boosting with the same immunogen can expand subdominant responses. Given that most HIV-1 vaccine trials have abbreviated vaccine regimens and contain a restricted Env sequence diversity compared to natural infection, it is unlikely that if and when a bNAb precursor is induced, there will be broad and potent neutralizing activity present in the plasma. The only way to determine if the vaccine-induced memory B-cell responses contain antibodies that target a vulnerable site on the virion and have the capacity to develop neutralization breadth and potency is by a deep and careful interrogation of the Env-reactive B-cell repertoire.

## ANTIBODY-DEPENDENT CELL-MEDIATED CYTOTOXICITY AND OTHER FC RECEPTOR-MEDIATED FUNCTIONS THAT HAVE PROTECTIVE EFFICACY

Although it is not yet possible to induce bNAb responses with vaccination, antibodies with limited neutralization breadth and with non-neutralizing Fc receptor (FcR)-mediated effector functions can be elicited. These types of antibodies have been correlated with reduced risk of infection during vertical transmission [42,43], in nonhuman primate challenge studies [44], and in the RV144 clinical trial [8,45,46]. In RV144, two immune correlates of decreased transmission risk were identified: high levels of V1/V2 binding antibodies, with subsequent analysis demonstrating a key role for antibodies specific to a linear V2 epitope centered on the lysine at amino acid position 169 (K169) in providing immune pressure that selected for virus escape [13,46]; and increased antibody-dependent cell-mediated cytotoxicity (ADCC) activity in the presence of low circulating Env-specific IgA [8,11^▪^]. Correlating with these observations, an unbiased analysis of the vaccine-induced memory B-cell repertoire identified V2-specific K169-dependent linear peptide-binding antibodies [46], and antibodies that target conformation epitopes in the first conserved region (C1). The C1 antibodies preferentially used the VH1 gene family, and constituted the major component of the ADCC response [45]. The isolated V2 mAbs are presumed to be representative of the class of antibodies associated with reduced infection risk, and were demonstrated to be capable of multiple antiviral functions including autologous tier 1 neutralization, ADCC activity against tier 2 virus infected cells, and the ability to capture infectious virus [46]. The levels of circulating V2 antibodies in vaccine recipients were not high enough to be effective based on in-vitro testing, but it was demonstrated that the anti-C1 and V2 antibodies could synergize to mediate ADCC at levels compatible with those present in vaccinees [47]. In contrast, a RV144 vaccine-induced IgA mAb was able to block the ADCC activity of vaccine-induced IgG antibodies [33]. Collectively, these observations generated the hypothesis that Fc-dependent immune functions including ADCC likely contributed to the protection observed for RV144, and high levels of IgA may have limited the vaccine efficacy through competition with beneficial antiviral functions of IgG.

The results of the RV144 correlates study highlighted the potential utility of non-neutralizing antibodies in prevention of HIV, leading to interest in exploring ADCC antibodies as immunotherapeutics for treatment and possible cure of HIV infection. Non-neutralizing antibodies including the C1 mAb A32 have recently been engineered into bi-specific antibody-based molecules that allow redirection of endogenous polyclonal CD8^+^ T cells for potent killing of HIV-infected cells and reactivated latently infected cells [48,49]. A phase I clinical trial is being planned to evaluate one of these bi-specific molecules in HIV-infected humans, and other novel therapeutics based both on ADCC antibodies and bNAbs are currently being tested in preclinical studies [50^▪▪^].

Collectively, the data from RV144 suggest that in absence of bNAbs, protection can be mediated by a polyclonal and polyfunctional antibody response. However, the RV144 study was conducted in a cohort with low risk of HIV infection [51]. It is unclear if the efficacy observed for RV144 would translate to higher-risk populations. Results from a high-dose mucosal challenge study conducted in nonhuman primates failed to demonstrate protection by an ADCC antibody, although the number of founder viruses that established infection were reduced compared to controls [52]. In a recent vertical transmission study, ADCC activity of passively acquired antibodies did not correlated with transmission risk, but higher ADCC was associated with reduced risk of infant mortality [53]. These data suggest that a vaccine with efficacy beyond that observed for RV144, and effective in higher-risk groups, will likely need to induce both neutralizing and non-neutralizing antibody responses working in concert to prevent infection, and possibly to control viremia and limit disease progression in the event of a breakthrough infection (contributions of C. Moog [12] and R. Ruprecht [54]).

## IMMUNIZATION IN INFANCY TO INDUCE BROAD NEUTRALIZATION BEFORE SEXUAL DEBUT

Inducing bNAbs will likely require novel immunization strategies that selectively recapitulate the immunological milieu in chronic HIV-1 infection. Potential strategies being explored include the use of novel immunogens based on structural understanding of the native envelope trimer and soluble stable trimers [55–59] (contribution of M. Ramirez [60]), B-cell lineage immunogen design [61,62], use of novel adjuvants (see review by J. McElrath [63]) to induce robust T-follicular helper cell responses [64,65^▪▪^], and the inclusion of immunomodulators to transiently inhibit immune tolerance checkpoints in order to overcome constraints that limit the development of polyreactive or autoreactive bNAbs [29^▪▪^]. Many of these candidate strategies will require several immunizations over an extended period of time. Therefore, immunization in infancy could be an attractive strategy to achieve durable broad anti-HIV neutralizing antibody responses prior to sexual debut. Although it is generally assumed that the developing infant immune system responds poorly to vaccination, recent studies have demonstrated that infants can mount robust responses following HIV vaccination [66^▪^]. Moreover, recent studies have indicated that young children can develop bNAb responses earlier than adults, and with neutralization breadth comparable to the top 1% of adult neutralizers [67]. Similarly, Adland *et al.* [68] measured neutralizing antibody responses in HIV-1-infected children and found that 70% of infant slow progressors, but only 15% of HIV-1 clade C chronically infected adults, were able to neutralize at least 50% of viruses tested. Children also had higher neutralization titers than adults. Importantly, Simonich *et al.* [69^▪^] reported that a broad neutralizing mAb isolated from a HIV-infected child has lower levels of mutation when compared to adult bNAbs, further suggesting that it could be easier to induce bNAbs in children than in adults. It is therefore critical to test novel immunization strategies in pediatric populations.

## CONCLUSION

All the HIV vaccine trials conducted to date, including the moderately efficacious RV144 Thai trial, failed to induce plasma bNAb responses. Recent analysis of the vaccine-induced memory B-cell repertoire in RV144/305 vaccine recipients demonstrated that repetitive immunization can shift the Env-specific repertoire expanding subdominant populations of B cells. These data highlight the importance of analyzing vaccine-elicited antibody responses beyond plasma. A thorough analysis of vaccine-elicited responses at the B-cell repertoire level is crucial to determine if a candidate vaccine was able to recruit subdominant B-cell lineages with characteristics of bNAbs, and to inform rational design of a boosting immunogen to select for B-cell clonal lineage members with increased neutralization breadth. Ultimately, an effective HIV vaccine will likely need to elicit a diverse antibody response that includes bNAbs and non-neutralizing antibodies with FcR-mediated antiviral effector functions.

## Acknowledgements

*We thank Drs Barton F. Haynes, Georgia D. Tomaras, Sallie R. Permar, and Guido Ferrari for helpful discussion in preparation of the manuscript.*

### Financial support and sponsorship

*The study was supported by the Center for HIV/AIDS Vaccine Immunology-Immunogen Discovery (CHAVI-ID; UM1-AI100645), the Duke University Center for AIDS Research (CFAR; NIH 5P30 AI064518), the University of North Carolina Collaboratory of AIDS Researchers for Eradication (CARE; U19 A1096113), by NIH Grants R21 AI127022 and R03 HD085871–01, Duke CTSA KL2 career development award (KL2TR001115), Duke Surgery Clarence E. Gardner award, and Collaboration for AIDS Vaccine Discovery grants from the Bill and Melinda Gates Foundation.*

### Conflicts of interest

*There are no conflicts of interest.*

## REFERENCES AND RECOMMENDED READING

Papers of particular interest, published within the annual period of review, have been highlighted as:

▪ of special interest
▪▪ of outstanding interest
